# Phenotypes, Risk Factors, and Mechanisms of Adult-Onset Asthma

**DOI:** 10.1155/2015/514868

**Published:** 2015-10-11

**Authors:** Pinja Ilmarinen, Leena E. Tuomisto, Hannu Kankaanranta

**Affiliations:** ^1^Department of Respiratory Medicine, Seinäjoki Central Hospital, 60220 Seinäjoki, Finland; ^2^Department of Respiratory Medicine, University of Tampere, 33014 Tampere, Finland

## Abstract

Asthma is a heterogeneous disease with many phenotypes, and age at disease onset is an important factor in separating the phenotypes. Genetic factors, atopy, and early respiratory tract infections are well-recognized factors predisposing to childhood-onset asthma. Adult-onset asthma is more often associated with obesity, smoking, depression, or other life-style or environmental factors, even though genetic factors and respiratory tract infections may also play a role in adult-onset disease. Adult-onset asthma is characterized by absence of atopy and is often severe requiring treatment with high dose of inhaled and/or oral steroids. Variety of risk factors and nonatopic nature of adult-onset disease suggest that variety of mechanisms is involved in the disease pathogenesis and that these mechanisms differ from the pathobiology of childhood-onset asthma with prevailing Th2 airway inflammation. Recognition of the mechanisms and mediators that drive the adult-onset disease helps to develop novel strategies for the treatment. The aim of this review was to summarize the current knowledge on the pathogenesis of adult-onset asthma and to concentrate on the mechanisms and mediators involved in establishing adult-onset asthma in response to specific risk factors. We also discuss the involvement of these mechanisms in the currently recognized phenotypes of adult-onset asthma.

## 1. Introduction

During the last decade, asthma has been revealed as a heterogeneous disease manifesting in many distinct phenotypes. Age at asthma onset has emerged as a critical factor in distinguishing these phenotypes. Patients with early-onset asthma are typically atopic with family history of atopy or asthma, Th2-predominant inflammation, good responsiveness to glucocorticoids, and good prognosis [1, 2]. In contrast, patients with adult- or late-onset asthma are most often nonatopic females without a family history of asthma or atopy and with less favourable prognosis and are more likely to develop persistent airflow limitation [3–8]. Even though majority of asthma is thought to be developed during childhood, this has been challenged recently by showing that, in the United States, adult-onset asthma is the dominant phenotype in women from 40 years of age [9].

Factors predisposing to adult-onset asthma include female sex, obesity, occupational exposure, rhinitis, respiratory infections, smoking, stressful life events, and low level of lung function [10–13] suggesting that adult-onset asthma may develop through a variety of mechanisms. This review aims to summarize the current knowledge on the pathogenesis of adult-onset asthma, concentrating on the known risk factors and on the mechanisms of how these factors might be involved in establishing asthma. We discuss the differences in the pathogenesis of adult-onset when compared to childhood-onset disease. We start by combining the information on cluster analyses identifying adult-onset asthma phenotypes, to enable association of the pathogenetic mechanisms with phenotypes, if possible.

## 2. Phenotypes of Adult-Onset Asthma

**Figure 1:**
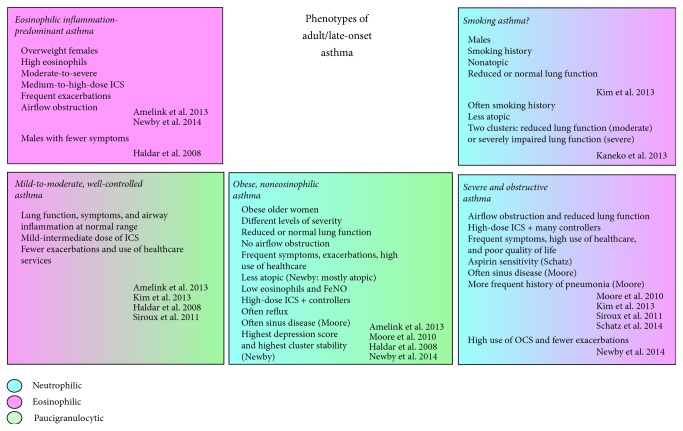
Currently identified phenotypes of adult/late-onset asthma based on published cluster analysis studies. ICS = inhaled corticosteroid, NSAID = nonsteroidal anti-inflammatory drug, OCS = oral corticosteroid, and FEV1 = forced expiratory volume in 1 second.

By combining information from cluster analyses concentrating on patients with adult-onset asthma [3] and of those including also patients with childhood-onset asthma [14–19], at least five different subtypes of late- or adult-onset asthma could be extracted (Figure 1 and Table 1). Even though plenty of resemblance was found regarding a phenotype obtained by different studies (e.g., obesity or eosinophil-predominant inflammation), also differences existed, reflecting most likely diversity of the study populations and techniques used, for example, differences in ethnicity, disease severity, method of recruitment, variables available, and variables included in the analysis. Whenever BMI was included as an input variable in cluster analysis, an obesity-related group was extracted, with the exception of Asian patient populations, where obesity is rare [14, 20]. Also exclusion or inclusion of smokers creates heterogenic results. Prevalence of smoking is generally high in many Asian populations and inclusion of smokers was nonrestricted in the two Asian analyses. A “smoking asthma” cluster was identified in a Korean analysis [14], whereas two clusters with higher rates of smoking were identified in a Japanese analysis (severe and moderate disease) [20]. The patients with moderate asthma were speculated to be more resistant to the effects of smoking [20]. Inclusion of smokers was limited in most US and European analyses, and thus “smoking asthma” clusters could not be identified.

Aspirin sensitivity was included as a separate cluster-defining variable in only two studies [3, 18] and the interpretation of the result is complicated by different patient selection. In patients with adult-onset asthma, only prevalence of nasal polyps but not nonsteroidal anti-inflammatory drug (NSAID) sensitivity was higher in patients with severe asthma when compared to milder disease [21]. In cluster analysis of the same population NSAID sensitivity was most prevalent in the phenotype with the mildest disease [3]. In another study including patients with difficult-to-treat asthma, aspirin-sensitive asthma was clearly extracted as its own phenotype, containing 58% patients with adult-onset asthma, and showed the highest risk for exacerbations and poor control [18]. In a further cluster analysis of the Severe Asthma Research Network (SARP) data including expanded lung data, a phenotype was separated with mostly late-onset asthma, strong history of nasal polyposis, sinusitis, blood and bronchoalveolar lavage (BAL) eosinophilia, increased blood neutrophil count, poor lung function, and high-dose corticosteroid use [22]. Whether nasal polyps are actually a more separating factor than aspirin sensitivity when identifying clusters remains to be determined. Also few smaller cluster analyses have been carried out, the results being complicated by lack of power [23, 24].

Biological mediators (other than IgE) have been rarely included in the cluster analyses published so far (Table 1), complicating the linking of clinical phenotypes to disease mechanisms. Mediators that could possibly be involved in adult- or late-onset asthma according to the limited evidence currently available are listed in Table 2, with association with a specific phenotype as suggested by the authors of this review. At the moment, periostin seems to be the most widely accepted biomarker. Generally, it is regarded as a biomarker of Th2-associated airway inflammation and predictor of airway eosinophilia and may predict response to anti-IL-13 and anti-IgE antibodies [25]. However, when studied in a population with severe asthma with majority having late-onset disease, it did not differentiate Th2-high and -low asthma but was raised in patients with eosinophilic airway inflammation when compared to mixed granulocytic, and in those with fixed airway limitation [26]. Thereby, in adult-onset asthma it could be used as a biomarker of eosinophilic inflammation-predominant asthma (Table 2).

## 3. Risk Factors for Adult-Onset Asthma

### 3.1. Obesity

Obesity-related late-onset phenotype arose in four different cluster analyses (Figure 1). Obesity is a risk factor for adult-onset asthma in both women and men, increasing the risk for asthma by approximately 50% [11, 27]. The association was stronger in nonallergic than allergic individuals [28]. Since obese patients typically consume high-fat diet, possess systemic inflammation, metabolic syndrome, and comorbidities, and breathe at lower lung volumes [29], it has been a topic of interest which of these is actually the predisposing factor for asthma. Very recently, it was suggested that peripheral lung might be inherently more collapsible in nonallergic obese females who develop late-onset asthma when compared to obese females who do not develop asthma; this was concluded from the more pronounced effects that weight loss had on lung elastance in patients with asthma [30]. BMI has been found as a better predictor of adult-onset asthma than metabolic syndrome in women [31], but insulin resistance was reported as a better predictor of asthma-like symptoms than BMI regardless of sex [32]. The possible common pathogenetic mechanisms of asthma and comorbidities traditionally nonrelated to asthma remain to be described.

It seems that there exist two types of obesity-related asthma; early-onset obese asthma is often not developed following obesity but rather complicated by obesity. Late-onset obese asthma is more often developed following obesity [33]. Both types are characterized by increased severity with increased BMI and high use of healthcare services despite use of high dose of ICS, suggesting glucocorticoid insensitivity [3, 15, 16, 33]. Obese patients with late-onset asthma were less atopic and had less bronchial hyperresponsiveness, less airway obstruction, fewer exacerbations, lower FeNO, but no difference in sputum eosinophilia or systemic markers of inflammation when compared to the early-onset group [33, 34].

Heterogeneity exists regarding airway inflammation in obese asthmatics [15, 34]. Studies with late-onset asthma as well as majority of other studies suggest low eosinophilia in obese asthma [33–38], even though one study with bronchial biopsy samples from obese severe asthmatics suggested only redistribution of eosinophils into airway submucosa [39]. In a recent cluster analysis, two clusters consisted mostly of obese patients with late-onset asthma and their inflammatory profiles were largely classified as neutrophilic or mixed granulocytic [40]. Also absence of airway inflammation (based on bronchial biopsy specimen) in obese patients with mild-to-moderate asthma was reported recently and no difference was observed between adult- and childhood-onset asthma [41].

One hallmark of obesity is a systemic low-grade inflammation with increased levels of many inflammatory markers such as C-reactive protein (CRP), interleukin- (IL-) 6, tumor necrosis factor- (TNF-) *α*, and leptin, and this is maybe important for the pathogenesis of obesity-related asthma [34, 42–45]. Systemic inflammation is generally considered to be a consequence of hypertrophy of adipocytes and their enhanced metabolic activity and macrophage infiltration into the adipose tissue at obese state [46]. Adipose tissue macrophages are polarized towards the classically activated proinflammatory M1 type (in contrast to anti-inflammatory M2 macrophages involved in tissue repair) [47]. In obese patients with asthma, increased number but reduced function (efferocytosis) of airway macrophages was shown, as well as reduced M2 marker expression in blood monocytes. Oxidative stress was found as a possible mechanism for altering macrophages [48]. Elevated systemic IL-6 and CRP as well as systemic biomarker of macrophage activation (soluble CD163) have been associated with poorer lung function and neutrophilic inflammation [49–51]. Also levels of YKL-40 were found highest in obesity-related phenotype (even though lowest in late-onset nonatopic asthma). YKL-40 was associated with poor asthma control and exacerbations, even though its function remains unclear [52] (Table 2).

Adipokines involve mediators with both proinflammatory (e.g., leptin and resistin) and anti-inflammatory (e.g., adiponectin) functions. Serum levels of leptin increase while the levels of adiponectin decrease with increasing BMI [53–57] but leptin has not been consistently shown to be increased in childhood- or adult-onset asthma when adjusted to BMI [58–61]. Leptin levels are higher in women with equivalent BMI when compared to men [62], and the association between leptin and asthma seems to be stronger in women [63]; both issues support the role of leptin in the pathogenesis of asthma in obese females. In nonobese women with adult-onset asthma, leptin correlated positively with asthma symptom score and negatively with lung function when adjusted for BMI [58] (Table 2). Inconsistent results have been reported on correlation between plasma and BAL leptin levels in patients with adult-onset asthma and no correlation regarding adiponectin [53, 56, 64]. Both clinical and experimental evidence suggests that leptin might rather function by augmenting airway hyperresponsiveness than by affecting inflammation [53, 65, 66] (Table 2). Leptin and adiponectin may have direct effect on airway hyperresponsiveness via their receptors in the airway epithelium and smooth muscle cells [53, 67, 68]. This is supported by a report of increased expression of their receptors in epithelia of obese asthmatics when compared to obese controls [53]. Furthermore, a significant negative correlation was found between visceral fat leptin expression and airway hyperreactivity to methacholine in patients with adult-onset asthma [53]. In contrast, a proinflammatory role in promoting eosinophilia is suggested by the finding that leptin acts as eosinophil survival-promoting factor [69, 70]. Adiponectin may act in different manner in women and men; in women, low serum adiponectin was associated with higher incidence of adult-onset asthma whereas men with higher serum adiponectin were associated with worse symptoms and more active disease [71, 72]. The association between asthma and adiponectin remains, altogether, inconsistent [63, 64].

Neutrophilic airway inflammation may be promoted by high-fat meal, suggesting involvement of this mechanism in obesity-related asthma. Obese and nonobese patients with asthma showed increased percentage of sputum neutrophils, expression of Toll-like receptor 4 (TLR4) mRNA, and suppressed response to bronchodilator after a meal with high content of fat or trans-fat [45]. Interestingly, this may be a more important route in males as compared to females, since the level of saturated and monounsaturated fatty acids predicted sputum neutrophil percentage in asthmatic males, but not females [73]. Indeed, free fatty acids have been shown to activate TLR4 pathway and innate immune response, constituting a mechanism for this phenomenon [74].

One attempt to the pathobiology of obesity-related adult-onset asthma was carried out recently by Holguin and coworkers. An inverse relationship between BMI and FeNO was observed in late- but not early-onset asthma. To explain the difference, they showed increased level of asymmetric dimethyl arginine (ADMA, an inhibitor of all NOS isoforms) and decreased ratio of L-arginine/ADMA in obese patients with late-onset asthma, which was less evident in early-onset asthma [75] (Table 2). The decreased ratio was also associated with worse outcome of asthma in late- but not early-onset asthma. Increased ADMA may direct iNOS to form superoxide instead of NO and on the other hand, since NO acts as bronchodilator [76, 77], reduced production of NO by iNOS could play a role in the pathogenesis of adult-onset obesity-related asthma. Reduced L-arginine and NO also suggest increased activation of arginase-polyamine pathway which may play a role in the pathobiology of asthma by promoting airway hyperreactivity or eosinophilia [78, 79].

Because many players are involved in obesity, the etiology of obesity-related late-onset asthma is not simple and straightforward. Inherent abnormal lung mechanics, systemic inflammation, and direct effects of adipokines on airway hyperresponsiveness and inflammatory cells may be involved, as well as mechanisms yet unexplored. Because majority of patients with adult-onset asthma are not only obese but also of female gender, the role of female sex hormones should be considered.

### 3.2. Gender and Sex Hormones

After puberty, females are clearly more often affected with asthma and have more severe disease [80–82]. Even though a smaller airway calibre may provide a partial explanation, evidence exists of the involvement of hormones in the disease pathogenesis. In prospective cohort studies, the risk of asthma in females has been reported to generally decrease after menopause, except in women using postmenopausal hormone replacement therapy [83, 84]. Current/recent use of oestrogen preparations increased risk of adult-onset asthma, while preparations containing both oestrogen and progestin showed contradictory results [83–85]. The risk was greatest amongst women who reported an allergic disease prior to asthma and in those who were never-smokers [83]. Smoking has antiestrogenic effects, which is one possibility to explain the reduced risk in smokers [85]. On the other hand, hormone replacement therapy was shown to improve the course of asthma in women with asthma [86]. Also phase of menstrual cycle has been reported to be associated with respiratory function, respiratory symptoms, and atopy in women with and without asthma, even though the results have been inconsistent [80, 87–89]. As suggested by differential risk of hormone-related adult-onset asthma between smokers and never-smokers, the effect of sex hormones on asthma risk or respiratory symptoms may differ in different subgroups of patients and explain the contradictory findings.

Th2-promoting capacity of estrogen on airway inflammation has been aroused in many mice studies [90–93], even though anti-inflammatory effects have been described as well [94]. The effects of progesterone have been less clear and are less studied [95, 96]. Similar findings have been obtained by using human cells [97, 98]. Testosterone, instead, has been shown to suppress Th2 cytokine production and increase IL-10 via androgen receptor of CD4+ T cells [99–101]. Neutrophils and whole blood cells from healthy males were also shown to produce smaller amount of 5-lipoxygenase (5-LO) products, leukotriene B4, and 5-hydroperoxyeicosatetraenoic acid (HPETE) when compared to female, because male androgens had a suppressive effect on the production [102]. These types of mechanisms may protect male from developing asthma after adolescence. Mice studies have also indicated that differences in the number of M2 macrophages in lung tissue after allergen exposure in female and male mice may lead to distinct regulation of airway inflammation [103]. Nonatopic males with adult-onset asthma were interestingly shown to exert an increased risk for persistent airflow limitation [6]. Smoking was not found to explain the finding, and most likely male hormones are not a significant player. For example, a common trigger of asthma in these subjects may be involved in the mechanism.

Nuclear receptors for sex hormones are expressed in healthy human lung tissue enabling direct effects on airway cells [104–106]. Even though the main mechanism of action of sex hormones is considered to be regulation of gene expression, they produce also nongenomic effects [107]. The nongenomic effects involve G-protein coupled estrogen receptor- (GPER-) mediated effects, modulation of ion channel function, and kinase activities [108–110]. Interestingly, a link between female sex hormones and eosinophils seems to exist, supported by the findings where eosinophils are recruited to female reproductive tissues with a mechanism related to estrogen [111–113]. Peripheral blood eosinophils were recently shown to express GPER and activation of this receptor enhanced chemotaxis in the presence of eotaxin (chemotactic agent for eosinophils) and modulated eosinophil viability [109], suggesting a direct mechanism for interaction between estrogen and eosinophils. Increased levels of eotaxin-2 have been found in bronchial epithelial brushings from patients with severe late-onset asthma, as well as its correlation with sputum eosinophilia [114] (Table 2). Whether estrogen further augments the chemotaxis of eosinophils into the airways in these patients* in vivo* remains to be determined. Given that free biologically active 17-*β* estradiol was higher in overweight when compared to normal-weight women, this type of eosinophilia-promoting mechanism may be present in overweight females, or those with otherwise high levels of estrogen [115]. Indeed, adult-onset asthma patients with severe eosinophil-predominant inflammation were mainly overweight females [3] (Figure 1). Obesity-related asthma is mainly considered as noneosinophilic, and obesity rather characterized by decreased level of estradiol [116], and thus eosinophilia-promoting capacity of estrogen is an unlikely mechanism to contribute to the phenotype.

The interplay between structurally similar sex hormones and corticosteroids and their effects on the modulation of airway inflammation is also an interesting issue that may affect development or severity of asthma [110]. Estrogen may, for example, inhibit production and function of cortisol [117] contributing in this manner to severe asthma.

### 3.3. Psychosocial Factors

Depressive disorders are at least twice as common in patients with asthma when compared to the general population [118, 119]. Psychosocial factors, such as perceived stress, childhood adverse events, early- and late-onset depression, and high extroversion score in women, have been reported as risk factors for adult-onset asthma [12, 120–124], even though the direction of causality between psychosocial factors and asthma still remains unclear and requires further studies. An association has also been shown, where patients with worse control of asthma showed increased risk of depression [118]. Depression may have common pathophysiological features with asthma explaining their coexistence but alternatively, stress of a chronic illness or treatment for it (long-term steroid treatment) may induce depression. Also common comorbidities and environmental factors, such as obesity and smoking, have been suggested to explain the association between depression and incident asthma but this hypothesis is not supported by the finding that the risk exists even after excluding these subgroups [12]. In a very recent cross-sectional study, depression and increased BMI were both associated with worse asthma control in adults but interestingly, depression was found to mediate the association between BMI and asthma control [125].

Several common pathophysiological pathways have been suggested to explain cooccurrence of asthma and major depressive disorder (MDD). In a recent meta-analysis, levels of IL-1, TNF-*α*, IL-6, and IL-4 were found higher in depressed patients when compared to nondepressed subjects [126] suggesting involvement of inflammatory pathways in the pathophysiological processes of MDD. These cytokines are elevated in at least specific phenotypes of asthma [34] and are able to generate symptoms such as fatigue and loss of appetite that overlap with symptoms of depression [127]. Cytokines may induce hyperactivation of hypothalamic-pituitary-adrenal (HPA) axis, ending up in increased cortisol levels and symptoms of depression [128]. Moreover, serotonergic neuron transmission is deficient in MDD and inflammatory cytokines elevate enzyme indoleamine-2,3-dioxygenase (IDO), which degrades tryptophan, the most important precursor of serotonin. IDO also increases kynurenine metabolites that have neurotoxic effects [129]. Biomarkers of oxidative stress are elevated in patients with MDD as well as patients with asthma. Reactive oxygen species (ROS) has been suggested directly, or via inducing inflammation, to damage cells and biomolecules, cause cell death and neurotoxic effects and reduce neurogenesis leading to MDD [130]. Additionally, cholinergic activation mediates airway constriction and has been associated with experience of hopelessness/depression suggesting that dysregulation of autonomous nervous system may be one mechanism in the pathogenesis of both diseases [128].

A recent hypothesis suggests that nod-like receptor protein 3 (NLRP3) inflammasome forms a link between stress, depression, and systemic disease [131]. NLRP3 inflammasome is a sensor for variety of danger substances ranging from pathogens (fungi, toxin-producing bacteria such as* S. aureus*, and viruses such as* H. influenza*) to elevated extracellular glucose, amyloid-*β* peptide, oxidized low-density lipoprotein (LDL), and number of environmental irritants [132]. NLRP3 inflammasome activates caspase-1 resulting in cleavage of pro-IL-1*β* into IL-1*β*. Psychological stressors have been shown to elevate IL-1*β* and may therefore activate inflammasome, even though direct mechanism has not been demonstrated [131]. Interestingly, in mice IL-1*β* and TNF-*α* also upregulated serotonin transporter (SERT) gene, and SERT by uptake of serotonin to the presynaptic neuron is central in inducing despair-like behaviour [133–135].

Clinical trials have provided further proof of an association between inflammatory and depressive disorders. Evidence exists that treatment with antidepressants (tricyclic/SSRIs) normalizes the levels of inflammatory cytokines in depressed patients [136]. Additionally, anti-inflammatory drugs have shown beneficial effects in the treatment of depression [131]. For example, treatment of patients with MDD with both antidepressant and NSAID celecoxib showed greater improvement in depressive symptoms when compared to treatment with antidepressant alone [137]. Altogether, a link has been constituted between depression, asthma, and inflammation, not forgetting obesity as a player in the pathophysiological process. In the cluster analysis of Newby et al. the highest depression score was present in the obesity-related phenotype [17] (Figure 1), strengthening the interplay between obesity, asthma, and depression. The causal relationships, however, require further studies.

### 3.4. Rhinitis, Sinusitis, and Respiratory Tract Infections

Rhinitis and sinusitis are frequently associated with asthma, regardless of age of onset. Rhinitis (allergic or nonallergic) is an independent risk factor for adult-onset asthma and the risk was further enhanced by belonging to the highest IgE tertile or by having a concomitant sinusitis [10, 138–140]. Chronic sinusitis alone, without clear nasal allergies, is very common in adult-onset asthma but markedly less prevalent in childhood-onset asthma [141]. Atopy seems to explain only minor portion of adult-onset asthma [10, 142]. One source of rhinitis and sinusitis is a respiratory tract infection, and consistently, recurrent infections of upper airways as well as infection of the lower airways were risk factors for adult-onset asthma. The risk was enhanced by current or past allergic rhinitis or atopic dermatitis or by an atopic parent [13]. The results are very similar to childhood asthma, where respiratory infections early in life have been shown to increase risk of asthma in childhood and to act synergistically with allergic sensitization [143].

Coexistence of upper and lower respiratory diseases and many shared morphological and functional properties of the upper and the lower respiratory tract suggest common underlying pathophysiological processes and have led to the theory of united airways diseases [144]. The close relationship between upper and lower airway diseases has also been shown in patients with adult-onset asthma. For example, sinus abnormalities are present in majority of patients with severe asthma, even in the absence of nasal symptoms, and were particularly associated with adult-onset asthma [145]. The degree of sinus disease positively correlated with eosinophilic airway and systemic inflammation and airway trapping and negatively with diffusion capacity in patients with adult-onset disease [145]. It was concluded that, in adult-onset severe disease, airway parenchyma and peripheral airways might participate in the disease progress, and inflammation might reach the alveolar wall. Nonallergic rhinitis is a general condition in patients with adult-onset asthma but it was shown that nonallergic and allergic patients with asthma possess strikingly similar nasal inflammation and nasal symptoms [146]. These “nonallergic” patients may therefore have a local allergic rhinitis, and local production of allergen-specific IgE, without never developing into systemic allergic rhinitis [147]. Thus, the close connection between upper and lower respiratory disease is most likely present in patients with adult-onset asthma, with similarities in the pathogenetic mechanisms.

Sometimes sinus disease/rhinitis and asthma are accompanied by aspirin sensitivity with or without nasal polyps, the clinical entity being named aspirin-exacerbated respiratory disease (AERD). Typically, AERD is an adult-onset disease, starting around 30 years of age with rhinitis, followed by asthma, aspirin sensitivity, and often nasal polyps. It is often more severe and more prevalent in females [148]. AERD is characterized by eosinophilic inflammation, overproduction, and responsiveness to cysteinyl leukotrienes and underproduction and responsiveness to prostaglandins in the airway inflammatory cells. These phenomena are augmented by aspirin and other NSAIDs, and, for example, alcohol, all inhibitors of cyclooxygenase- (COX-) 1 enzyme, which further moves the balance between synthesis of prostanoids and cysteinyl leukotrienes towards the latter [149, 150]. Recently, it was demonstrated that AERD patients show high levels of both IL-4 and IFN-*γ* in sinus tissue, suggesting a mixed Th1/Th2 milieu instead of solely Th2. Eosinophils were the main source of IFN-*γ*, and this cytokine was able to promote maturation and cysteinyl leukotriene production of eosinophils [151]. Also patients with severe poorly controlled late-onset asthma showed enhanced IFN-*γ* and IL-8 when compared to moderate disease, showing further proof that certain phenotypes of adult-onset disease may have a significant Th1 component (Table 2) [152]. Also variants of genes related to arachidonate pathway, inflammation, and immune responses [153, 154] as well as staphylococcal superantigens [155] may play a role in the pathogenesis of AERD. Patients with AERD (majority of them with adult-onset disease) were associated with fewer comorbidities in general (e.g., components of metabolic syndrome) but more coronary heart disease or congestive heart failure when compared to asthma without AERD [156]. Whether a common pathogenetic component exists remains to be studied.

*Staphylococcus aureus *is a bacterium responsible for many infections (e.g., sinusitis) but may also be commensal. Serum IgE specific to* S. aureus *enterotoxin (SA-IgE) has been linked to adult-onset asthma and worse outcome of asthma [157–159]. IgE may be formed against bacterial/viral components or products in both atopic and nonatopic patients and presence of pathogen-specific IgE may reflect current or past contact with the pathogen.* S. aureus *enterotoxin may act as antigen promoting a specific IgE response (SA-IgE) or as superantigen stimulating massive activation of lymphocytes, formation of polyclonal IgE (reflected as high total IgE), and Th2 response [157, 160]. In a recent study, approximately 60% of adults with severe asthma were positive for SA-IgE, majority being nonatopic [157]. SA-IgE positivity in nonatopic patients was associated with increased use of oral steroids and hospitalizations, lower FEV1, and disease onset at higher age [157]. Among patients with adult-onset asthma the presence of SA-IgE was associated with male gender, current smoking, age ≥61 years, and inhalant allergen sensitization and marginally with diabetes mellitus [158] (Table 2). Functional SA-IgE was also detected in polyp tissue from subjects with nasal polyps; presence of IL-5 and SA-IgE was associated with comorbid asthma [161, 162]. In addition to markers of eosinophilic inflammation, these patients showed high systemic total IgE (>450 kU/L) [161].* Staphylococcus aureus *enterotoxin-driven massive IgE production and Th2 inflammation may be one mechanism explaining the overlap between severe asthma, rhinitis, and/or nasal polyps [157, 159, 161].

History of sinusitis and pneumonia is more common among patients with severe late-onset asthma when compared to severe early-onset asthma. Pneumonia was also a strong predictor of severe asthma, suggesting that pathogens causative for pneumonia may be involved in the pathogenesis of the disease [163].* Chlamydophila pneumoniae *and* Mycoplasma pneumoniae* are bacteria causing pneumonia and many other infections of upper and lower respiratory tract and have been linked to both adult- and childhood-onset asthma [164–167]. In a Finnish longitudinal study, seropositivity for* C. pneumoniae* was not a risk factor for adult-onset asthma [168], even though it has been associated with asthma or asthma severity in several studies including patients with adult-onset asthma [164, 166, 167]. Seropositive patients with nonatopic adult-onset asthma had significantly steeper decline in lung function when compared to seronegative patient groups or seropositive patients with early-onset asthma [7, 168] (Table 2). Additionally,* C. pneumoniae*-specific IgE was associated with disease severity in population of asthmatic patients, where half had disease onset in adulthood [164] (Table 2). Age of onset did not affect the likelihood of being seropositive among patients with severe asthma. Seropositive patients with nonatopic adult-onset asthma used high doses of inhaled and/or oral steroids suggesting that they were relatively insensitive to steroids and may require alternative treatment options [7].* C. pneumoniae* could function by activating* C. pneumoniae*-specific IgE/Fc*ε*R1 complex on mast cells or basophils, enhancing Th2 inflammatory response [164]. It may also contribute to airway remodelling by enhancing production of several growth factors and cytokines by the airway structural cells [169–171]. Furthermore,* C. pneumoniae* may alter cellular responsiveness to glucocorticoids [172] explaining the need for high steroid doses of seropositive patients. The possible role of* C. pneumoniae *in the etiology or progression of coronary heart disease in addition to asthma [173] raises interesting possibilities for common pathogenetic or disease-modifying factors in these diseases.

It has been postulated that chronic diseases of the respiratory tract including asthma, rhinitis, and sinusitis are all manifestations of defective mucosal function, suggesting that the primary causal phenomenon would be the defective epithelial/mucosal function leading to higher susceptibility to respiratory tract infections and asthma. Thereby, the direction of causality remains unclear. Patients with asthma have disrupted epithelial cell tight junctions in the airways enhancing passage of antigens and they are more susceptible to the proteolytic or prooxidative effects of allergens, respiratory viruses, air pollutants, and tobacco smoke [174–176]. Whether these defects are present before the disease onset remains unclear. Asthma susceptibility genes that encode proteins related to epithelial integrity or function have been associated with childhood-onset asthma [177, 178] and may be found in phenotypes of adult-onset asthma (severe adult-onset asthma, asthma-COPD overlap) [179, 180]. Even though the susceptible individual may be spared from the disease during childhood, changes in life-style or environmental factors could trigger asthma in adulthood. Also allergy or smoking may produce defects in the defence system against respiratory tract infections [181–183]. Allergic reaction and IgE crosslinking with its receptor (Fc*ε*R1) in plasmacytoid dendritic cells prior to infection may lead to insufficient production of IFN in response to viral infection [181, 184, 185]. This may promote a prolonged, more severe infection leading more likely to a chronic airway disease [186]. Mechanism for the development of chronic airway inflammation in response to viral respiratory tract infection has been suggested based on an experimental study. Interestingly, the mechanism was based on an innate immune response. The acute response to viral respiratory tract infection was followed by a delayed response with IL-13 production and manifestation of airway hyperresponsiveness and mucous cell metaplasia. The cellular source of IL-13 was shown to be macrophages exhibiting markers of alternative activation (M2) and the production was driven by direct interaction of macrophages with natural killer T (NKT) cells. At the time of the delayed response, very low, almost undetectable level of the virus was present in the lungs. The mechanism had also a genetic aspect since another mouse strain did not develop a similar chronic airway inflammation [187]. Apart from immunological defects, also pathogen-recognition receptors such as Toll-like receptors (TLRs) and innate immune response may have importance in the development of asthma [174]. Interestingly, some allergens such as house dust mite (HDM) allergen, Der p, have been shown to mimic proteins required for Toll-like receptor 4 activation [188]. Stimulation by HDM led to TLR4 activation in structural airway cells and induced production of “proallergic” TSLP, GM-CSF, IL-25, and IL-33 and DC activation [189]. Thus, HDM by triggering an innate immune response initiated an allergic response. Bacterial and viral components may also directly activate and increase longevity of granulocytes via TLRs [190–192].

Alterations in the immune functions are manifested with increasing age and these changes most likely increase susceptibility of elderly to respiratory infections. Alterations seen in subjects above 60–65 years include reduced mucociliary clearance, reduced phagocytic capacity and increased apoptosis in neutrophils, decreased degranulation of eosinophils, and defects in antigen phagocytosis and presentation and lymphocyte function resulting in reduced levels of antibody production [193]. These changes may predispose older individuals to more severe infections and development of late-onset asthma.

### 3.5. Alcohol

In a large Danish twin-study, overall alcohol intake was associated with the risk of adult-onset asthma in a U-shaped manner. Subjects with moderate weekly intake (1–6 units/week) showed the lowest risk of incident asthma, while the highest risk was observed in the group of rare/never drinkers [194]. When the biomarkers of alcohol consumption were studied, mathematical combination of carbohydrate-deficient transferrin (CDT) and *γ*-glutamyltransferase (GGT) was positively associated with self-reported asthma in women but not men [195].

Alcohol has complex associations with asthma. Pure ethanol is a moderate and transient bronchodilator but nonalcoholic components of alcoholic beverages (e.g., sulphites of red wine) and acetaldehyde (product of ethanol metabolism) may act as triggers of asthma attacks [196]. Those who suffer from wine-induced symptoms are often women with early-onset asthma [197].

Alcohol consumption increases the level of serum total IgE, even when consumed at lower quantities (10–70 g/week) [198–200]. This effect was seen in both atopics and nonatopics [198, 200–202] but its clinical significance for IgE-mediated diseases remains unclear. Additionally, despite the elevated IgE levels, it is also unclear whether chronic alcohol abuse leads to Th2 predominance. Impaired Th1 cell-mediated immunity and polarization towards Th2 response [203, 204] but also Th1 predominance has been reported in alcoholics [198]. Duration and amount of alcohol consumption may play a pivotal role in determining its effect on inflammation. It was shown that moderate, acute alcohol use* in vitro* and* in vivo* resulted in anti-inflammatory effects on human monocytes whereas chronic use led to upregulation of NF-*κ*B and proinflammatory effects. Also activation status of the cells was important; higher activation state (the presence of costimulators) turned the effects of acute use into proinflammatory [203]. These interesting observations may reveal the basis for increased risk of asthma among heavy drinkers and the beneficial effects of moderate alcohol use.

### 3.6. Smoking and Oxidative Stress

The current evidence suggests, even though it is not conclusive, that active or passive smoking is a risk factor for adult-onset asthma [10, 205–208], the risk being greatest in individuals with allergic rhinitis [209]. Healthy smoking effect most likely contributes to the partially conflicting results; those with sensitive airways or any respiratory problems may not start smoking or quit more easily [210–213]. Smoking increases asthma severity [210, 214], and current evidence suggests that this effect of smoking also occurs in patients with adult-onset disease. A two-year follow-up of patients with new-onset adult asthma showed that history of smoking at baseline predicted increased asthma severity in a dose-dependent manner [215]. Smoking accelerated the normal annual decline in lung function in nonatopic patients with early- or late-onset asthma (onset ≥ 10 years) [216]. Current smoking also increased risk of airway obstruction but only in patients with late-onset asthma. The greatest risk was among those with asthma onset during adolescence, which is the time of maximal lung growth [216]. Another study showed that especially current smokers with asthma (early- or late-onset) and atopy are susceptible to fixed obstruction, with stronger association among patients with early-onset asthma [217]. In patients with mild newly diagnosed adult-onset asthma, the effectiveness of low dose ICS therapy in reducing FEV1 decline was not affected by smoking [218] even though the result might be different in patients with severe asthma.

Smoking increases oxidative stress and has proinflammatory effects on the lungs of nonasthmatics; these are changes predisposing for development of asthma. Smoking increases number of airway inflammatory cells (neutrophils, macrophages), as well as inflammatory cytokine production in nonasthmatics [219, 220]. Airway epithelia is in direct contact with cigarette smoke and produces IL-1*β* and IL-8 being responsible for the neutrophil recruitment [221]. Epithelium also undergoes many changes, such as disruption of tight junctions leading to increased permeability and reduced barrier function [182, 183], altered structure and function of mitochondria [222], changes in gene expression patterns [223, 224], and ageing [225] in response to cigarette smoke. In smokers without asthma, epithelial integrity was reduced and negatively correlated to number of eosinophils and macrophages, and thickness of the tenascin and laminin layers was increased [220]. Smoking patients with asthma exhibited similar changes in epithelia, as well as increased proliferation rate of epithelial cells, most likely to cope with the smoke-induced damage. Ex-smokers (at least one year without smoking) did not exhibit these changes in epithelium, suggesting that smoke-induced changes can be reversed by smoking cessation. Ex-smokers, however, still showed increased neutrophilic inflammation [226, 227].

One puff of cigarette smoke contains approximately 10^14^ oxygen radicals and 3000 ppm NO leading to increased oxidative stress in smokers. Airway epithelium is damaged and shed by oxidants. Oxidants impair cell membrane lipids, inactivate enzymes/receptors, oxidate/nitrosylate transcription factors and kinases leading to modified expression of inflammatory genes, and contribute to formation of other bioactive molecules such as 8-isoprostane (bronchoconstrictor) [228]. In addition to pathogenesis of smoking-related asthma, increased oxidative stress and reduced antioxidant levels may contribute to development of adult-onset asthma in general [228–230]. Thymic stromal lymphopoietin (TSLP) is one possible mediator of smoking-induced adult-onset asthma; it was elevated in the sputum of smokers with adult-onset asthma, positively correlated with pack-years and negatively correlated with FEV1/FVC [231] (Table 2). It is produced mainly by epithelial cells. Studies in mice also suggest involvement of TSLP in smoking-induced asthma [232].

### 3.7. Air Pollution

Increasing body of literature suggests, though it is not conclusive, that traffic-related air pollution (NO_2_ and particulate matter less than 2.5 *μ*m in diameter [PM_2.5_]) increases the risk for adult-onset asthma [233–235]. Most studies on air pollution and asthma have concentrated on children, but whether different mechanisms are involved in patients with adult-onset asthma who often are less atopic remains unclear. In elderly women, long-term exposure to traffic- and industrial-related air pollution was associated with increased inflammatory markers (leukotriene B_4_ and TNF-*α*) in exhaled breath condensate and induced sputum [236]. Oxidative stress has been hypothesized as the mechanism of how air pollution might cause asthma. NO_2_ is a free radical and specific components of PM_2.5_ also induce oxidative stress [235, 237]. Additionally, polymorphism of genes involved in the pathways of oxidative stress may affect susceptibility to asthma in response to air pollution [238]. However, the mechanisms as well as association between air pollution and adult-onset asthma are still uncertain and require further studies.

### 3.8. Occupational Exposures

Work-related asthma (occupational or work-exacerbated) is estimated to account for 10–25% of adult-onset asthma cases [239, 240]. Occupational asthma may be developed via several different mechanisms and should thus not be regarded as one single phenotype [241]. Additionally, methodological and legal aspects hamper specific definition of occupational asthma.

Occupational asthma is divided into sensitizer- and irritant-induced asthma. The causative agents of occupational asthma, high-molecular weight (HMW) proteins (e.g., from animals, plants, microorganisms) and low-molecular weight (LMW) chemical agents (e.g., toluene diisocyanate), seem mainly to use different mechanisms to develop asthma, IgE- and non-IgE-mediated mechanism, respectively. HMW factor-induced IgE-mediated asthma accounts for majority of occupational asthma [242, 243]. Specific IgE is rarely detected in asthma induced by LMW chemicals, and FeNO levels have been reported to be lower when compared to HMW factor-induced asthma; occupational asthma induced by LMW chemicals seems to constitute its own phenotype [241] with several speculated mechanisms [242]. Irritant-induced asthma develops after acute high exposure to vapor, gas, fume, or smoke. It is thought to develop following inhalation injury by a nonimmunological route but the pathogenetic process is mostly unknown [244]. More detailed discussion of mechanisms of asthma phenotypes related to occupational exposures, their prognosis, and treatment can be found elsewhere [240–242, 244–247].

## 4. Conclusions and Future Perspectives

Even though childhood- and adult-onset asthma may share some pathogenetic mechanisms, for example, those related to united airway diseases, obesity, and psychological distress, also significant differences exist. Factors such as hormones and those associated with life-style or work (alcohol, active smoking, and occupational exposure) of course mainly affect adolescence/adulthood and thereby only trigger or modify adult/adolescent-onset disease. However, also factors that are involved in both early- and late-onset diseases, such as seropositivity to* Chlamydophila pneumonia*, show striking differences to the disease progress depending on the disease onset and atopy status, suggesting that adult-onset disease has unique features.

So far, cluster analyses defining the phenotypes of asthma have been based mainly on clinical variables and less on biological markers. To what degree a common mechanism explains a specific phenotype identified based on clinical variables remains unknown. If endotypes and clinical phenotypes do not overlap with high extent, success of one therapy to treat all subjects inside a clinical phenotype is questionable. However, it is promising that identification of the clinical phenotypes of asthma has aided in revealing the genetic heterogeneity of the disease [248], suggesting that common genetic variants and thereby common mechanisms are involved in specific phenotypes. Use of biological markers in the cluster analyses would give us more detailed information on endotypes and disease pathogenesis and open possibilities for novel treatments. In addition, inclusion of major comorbidities traditionally nonrelated to asthma (e.g., psychiatric disorders, type II diabetes, and coronary heart disease) to cluster analyses may significantly affect the end result raising novel phenotypes. The coexistence of adult-onset asthma with many disorders suggests involvement of common pathogenetic mechanisms and is an interesting area for further studies. Whether the current clinical phenotypes are only preliminary or close to the final remains an open question. Because the current phenotypes (such as obesity-related ones) aroused in different studies have more similarities than differences, they are a good starting point for further analyses.

## Figures and Tables

**Table 1:** Background information of cluster analyses including characterization of phenotypes of adult-onset asthma.

Study	Patient *n*	Duration of asthma (mean/median years)	Patients with early-onset asthma included^*∗*^	Severity levels included	Major comorbidities (other than rhinitis/atopy) included as input or outcome variables	Smokers included	Other exclusion criteria	Study setting CS = cross-sectional L = longitudinal (time followed)	Variables related to biomarkers included in cluster analysis
Haldar et al. 2008 [16]	(a) 184 (b) 187 (c) 68	NR	Yes	(a) Mild-to-moderate, (b-c) refractory	(b) Scores for anxiety (input), depression and hyperventilation syndrome	Ex-smokers with <10 pack-years	(c) Therapy nonadherence	(a-b) CS (c) L (1 year)	Sputum eosinophil count

Moore et al. 2010 [15]	(d) 726	22	Yes	All	Aspirin sensitivity/polyps, pneumonia/bronchitis, sinus disease, GERD, and hypertension (all input, included in composite variables)	Ex-smokers with <5 pack-years	Age below 12 years	CS	None

Siroux et al. 2011 [19]	(e) 1895 (f) 641	NR	Yes	All	Eczema (input)	Yes	(e) Age below 20 years (f) Children	CS (population-based)	Total IgE

Amelink et al. 2013 [3]	(g) 200	10	No	All	NSAID sensitivity (input), history of nasal polyposis, and reflux symptoms/medication	Yes if ≥12% FEV1 reversibility after salbutamol inhalation and normal diffusion capacity.	Respiratory symptoms or chronic lung diseases during childhood, other pulmonary diseases, or nonrelated major comorbidities, pregnancy.	CS	Sputum eosinophils, total IgE

Kim et al. 2013 [14]	(h) 724 (i) 1843	NR	Yes	All	None	Yes	(h) Subjects with destroyed lungs, bronchiectasis or lung resection, age below 18 years (i) age below 18 years	(h) L (1 year) (i) CS	None

Newby et al. 2014 [17]	(j) 349	NR	Yes	Severe refractory	Scores for anxiety and depression, reflux history, nasal polyps, and eczema	Yes	NR	L (median 3 years)	Blood eosinophil count, total IgE

Schatz et al. 2014 [18]	(k) 3612	NR	Yes	Difficult-to-treat asthma (all)	Aspirin sensitivity, atopic dermatitis (input)	Yes, smoking history ≤30 pack-years	(k) Age below 12 years	L (1 year)	Total IgE

Kaneko et al. 2013 [20]	(l) 880	NR	Yes	All	None	Yes	NR	CS	Total IgE

NR = not reported, GERD = gastroesophageal reflux disease, NSAID = nonsteroidal anti-inflammatory drug, and FEV1 = forced expiratory volume in 1 second. (a), (b), and (c) Patients recruited from several different studies and sources (a = primary care, b = secondary care, c = data from a prospective clinical study), (d) SARP = the Severe Asthma Research Network, (e) ECRHSII = European Community Respiratory Health Survey, (f) EGEA2 = Epidemiological Study on the Genetics and Environment of Asthma, (g) patients recruited from hospitals in Amsterdam, (h) COREA = Cohort for Reality and Evolution of Adult Asthma in Korea, (i) SCH = Korean asthma cohort of Soonchunhyang University Asthma Genome Research Centre, (j) patients from British Thoracic Society Severe Refractory Asthma Registry, (k) TENOR = The Epidemiology and Natural History of Asthma: Outcomes and Treatment Regimens, and (l) patients recruited from University of Tsukuba Hospital, Hokkaido University Hospital, or their affiliated hospitals, Japan. ^*∗*^Defined as age at onset <18 years.

**Table 2:** Biological mediators and biomarkers suggested to be involved in adult/late-onset asthma.

Mediator	Level of asthma severity included	Inclusion of patients with adult/late-onset asthma	Main finding concerning the role of mediators in adult/late-onset asthma.	References	Adult/late-onset phenotype with possible significance
Periostin	Severe	80% with late-onset asthma	Higher levels in patients with fixed airflow limitation and eosinophilic inflammation.	Bobolea et al. 2015 [26]	Eosinophilic inflammation-predominant

Eotaxin-2	All	Groups of early- and late-onset asthma compared	Patients with severe asthma showed higher epithelial expression of eotaxin-2. Levels of eotaxin-2 positively correlated with age of asthma onset, sputum eosinophils, and number of exacerbations and negatively correlated with FEV1 % predicted. Among patients with late-onset (but not early-onset) asthma, eotaxin-levels were higher in those with severe asthma. Sputum eosinophilia was more common among patients with late-onset asthma.	Coleman et al. 2012 [114]	Eosinophilic inflammation-predominant

L-arginine/ADMA	Severe	Groups of early- and late-onset asthma compared	Lower L-arginine/ADMA ratio and negative correlation to BMI in late- versus early-onset asthma; may partly explain the inverse relationship between FeNO and BMI. Reduced L-arginine/ADMA ratio associated with reduced IgE and lung volumes, increased respiratory symptoms, and worse quality of life in late- but not early-onset asthma.	Holguin et al. 2013 [75]	Obesity-related asthma

Leptin	All	Obese women with adult-onset asthma	Leptin in visceral fat associated with AHR but not airway inflammation. Adipokine receptor was expressed in airway epithelium suggesting direct effects of adipokines on the airways.	Sideleva et al. 2012 [53] Leivo-Korpela et al. 2011 [58]	Obesity-related asthma
	All	Nonobese women with newly-onset adult asthma	Leptin correlated positively with asthma symptom score and negatively with lung function even though no difference was found on adipokine levels between patients with asthma and healthy subjects. High baseline resistin predicted steeper decrease in serum ECP, EPX, and MPO during glucocorticoid treatment.		

YKL-40	All	Late- and early-onset groups compared	Levels of YKL-40 were higher in asthma with poor control and exacerbations and atopy. When phenotypes were compared, highest levels of YKL-40 in obesity-related asthma and early-onset atopic asthma. Lower levels in nonatopic late-onset asthma.	Specjalski et al. 2015 [52]	Obesity-related (and early-onset atopic)

IFN-*γ* and IL-8	All	Patients with moderate and severe asthma compared, approx. 85% with asthma onset ≥12 years in both groups	Levels of IFN-*γ* and IL-8 increased but IL-4 decreased in airway subepithelium of patients with severe poorly controlled asthma when compared to moderate disease. Patients with severe asthma had also increased number of eosinophils and neutrophils in sputum.	Shannon et al. 2008 [152]	Severe and obstructive asthma?

*Chlamydia pneumonia* (IgG, IgA, and IgE)	All	Groups of child- and adult-onset asthma compared	Patients with nonatopic adult-onset asthma and seropositivity to *Chlamydia pneumoniae* (IgG, IgA) had 4-fold-steeper decline in FEV1/FVC and accelerated decline in FEV1. *C*. *pneumoniae* infection might promote persistent airflow limitation in these patients.	Brinke et al. 2001 [7]	Severe and obstructive asthma
	All	Patients with adult-onset asthma	Seropositivity to *C*. *pneumoniae* (IgG/IgM, IgA) infection was found to accelerate the loss of lung function in subjects with new nonatopic asthma.	Pasternack et al. 2005 [168]	
	All	52% with adult-onset asthma	*C. pneumoniae* IgE was detected in 21% of mild intermittent and 79% of severe persistent asthma. *C. pneumoniae* IgE was associated with asthma and asthma severity.	Hahn et al. 2012 [164]	

*Staphylococcus aureus* enterotoxin- (SA-) IgE	All	Healthy controls, severe and nonsevere asthmatics compared	SA-IgE found more often in patients with severe asthma when compared to healthy subjects. Age of asthma onset highest in SA-IgE-positive group. SA-IgE associated with increased risk for asthma, severe asthma, hospitalizations, use of oral steroids, and lower FEV1.	Bachert et al. 2012 [157]	Asthma in aged persons?
	All	Community-based population with adult-onset asthma	Risk factors for presence of SA-IgE were current smoking, older age, male sex, and inhalant allergen sensitization. SA-IgE was associated with current adult-onset asthma. Correlation between SA-IgE and total IgE.	Song et al. 2014 [158]	

TSLP	All	Patients with newly diagnosed asthma (age range 16–98 years)	Smoking attenuated the age-related decrease in total IgE, blood eosinophils, and FeNO and maintained eosinophilic inflammation. Sputum TSLP levels were associated with sputum eosinophil % and pack-years. Current and ex-smokers had higher TSLP versus never-smokers. TSLP may be involved in elevating sputum eosinophils in smokers.	Nagasaki et al. 2013 [231]	Smoking asthma

NR = not reported. ADMA = asymmetric dimethyl arginine, AHR = airway hyperresponsiveness, ECP = eosinophilic cationic protein, EPX = eosinophil peroxidase, MPO = myeloperoxidase, IFN = interferon, IL = interleukin, SA-IgE = *Staphylococcus Aureus* enterotoxin-specific immunoglobulin E, FEV1 = forced expiratory volume in 1 second, and TSLP = thymic stromal lymphopoietin.

## References

[1] Bisgaard H., Bonnelykke K. (2010). Long-term studies of the natural history of asthma in childhood. *Journal of Allergy and Clinical Immunology*.

[2] Paaso E. M., Jaakkola M. S., Rantala A. K., Hugg T. T., Jaakkola J. J. (2014). Allergic diseases and asthma in the family predict the persistence and onset-age of asthma: a prospective cohort study. *Respiratory Research*.

[3] Amelink M., de Nijs S. B., de Groot J. C. (2013). Three phenotypes of adult-onset asthma. *Allergy*.

[4] Wenzel S. E. (2012). Asthma phenotypes: the evolution from clinical to molecular approaches. *Nature Medicine*.

[5] Rönmark E., Lindberg A., Watson L., Lundbäck B. (2007). Outcome and severity of adult onset asthma—report from the obstructive lung disease in northern Sweden studies (OLIN). *Respiratory Medicine*.

[6] Amelink M., de Nijs S. B., Berger M. (2012). Non-atopic males with adult onset asthma are at risk of persistent airflow limitation. *Clinical and Experimental Allergy*.

[7] Brinke A. T., van Dissel J. T., Sterk P. J., Zwinderman A. H., Rabe K. F., Bel E. H. (2001). Persistent airflow limitation in adult-onset nonatopic asthma is associated with serologic evidence of *Chlamydia pneumoniae* infection. *Journal of Allergy and Clinical Immunology*.

[8] Tuomisto L. E., Ilmarinen P., Kankaanranta H. (2015). Prognosis of new-onset asthma diagnosed at adult age. *Respiratory Medicine*.

[9] Sood A., Qualls C., Schuyler M. (2013). Adult-onset asthma becomes the dominant phenotype among women by age 40 years. The longitudinal CARDIA study. *Annals of the American Thoracic Society*.

[10] Jamrozik E., Knuiman M. W., James A., Divitini M., Musk A. W. (2009). Risk factors for adult-onset asthma: a 14-year longitudinal study. *Respirology*.

[11] Ronmark E., Andersson C., Nystrom L., Forsberg B., Jarvholm B., Lundback B. (2005). Obesity increases the risk of incident asthma among adults. *European Respiratory Journal*.

[12] Brunner W. M., Schreiner P. J., Sood A., Jacobs D. R. (2014). Depression and risk of incident asthma in adults: the CARDIA study. *American Journal of Respiratory and Critical Care Medicine*.

[13] Rantala A., Jaakkola J. J. K., Jaakkola M. S. (2011). Respiratory infections precede adult-onset asthma. *PLoS ONE*.

[14] Kim T.-B., Jang A.-S., Kwon H.-S. (2013). Identification of asthma clusters in two independent Korean adult asthma cohorts. *European Respiratory Journal*.

[15] Moore W. C., Meyers D. A., Wenzel S. E. (2010). Identification of asthma phenotypes using cluster analysis in the severe asthma research program. *American Journal of Respiratory and Critical Care Medicine*.

[16] Haldar P., Pavord I. D., Shaw D. E. (2008). Cluster analysis and clinical asthma phenotypes. *The American Journal of Respiratory and Critical Care Medicine*.

[17] Newby C., Heaney L. G., Menzies-Gow A. (2014). Statistical cluster analysis of the british thoracic society severe refractory asthma registry: clinical outcomes and phenotype stability. *PLoS ONE*.

[18] Schatz M., Hsu J.-W. Y., Zeiger R. S. (2014). Phenotypes determined by cluster analysis in severe or difficult-to-treat asthma. *The Journal of Allergy and Clinical Immunology*.

[19] Siroux V., Basagan X., Boudier A. (2011). Identifying adult asthma phenotypes using a clustering approach. *European Respiratory Journal*.

[20] Kaneko Y., Masuko H., Sakamoto T. (2013). Asthma phenotypes in Japanese adults—their associations with the CCL5 and ADRB2 genotypes. *Allergology International*.

[21] Amelink M., de Groot J. C., de Nijs S. B. (2013). Severe adult-onset asthma: a distinct phenotype. *Journal of Allergy and Clinical Immunology*.

[22] Wu W., Bleecker E., Moore W. (2014). Unsupervised phenotyping of severe asthma research program participants using expanded lung data. *Journal of Allergy and Clinical Immunology*.

[23] Bourdin A., Molinari N., Vachier I. (2014). Prognostic value of cluster analysis of severe asthma phenotypes. *The Journal of Allergy and Clinical Immunology*.

[24] Sakagami T., Hasegawa T., Koya T. (2014). Cluster analysis identifies characteristic phenotypes of asthma with accelerated lung function decline. *Journal of Asthma*.

[25] Parulekar A. D., Atik M. A., Hanania N. A. (2014). Periostin, a novel biomarker of TH2-driven asthma. *Current Opinion in Pulmonary Medicine*.

[26] Bobolea I., Barranco P., Del Pozo V. (2015). Sputum periostin in patients with different severe asthma phenotypes. *Allergy*.

[27] Beuther D. A., Sutherland E. R. (2007). Overweight, obesity, and incident asthma: a meta-analysis of prospective epidemiologic studies. *American Journal of Respiratory and Critical Care Medicine*.

[28] Chen Y., Dales R., Jiang Y. (2006). The association between obesity and asthma is stronger in nonallergic than allergic adults. *Chest*.

[29] Ali Z., Ulrik C. S. (2013). Obesity and asthma: a coincidence or a causal relationship? A systematic review. *Respiratory Medicine*.

[30] Al-Alwan A., Bates J. H. T., Chapman D. G. (2014). The nonallergic asthma of obesity. A matter of distal lung compliance. *The American Journal of Respiratory and Critical Care Medicine*.

[31] Assad N., Qualls C., Smith L. J. (2013). Body mass index is a stronger predictor than the metabolic syndrome for future asthma in women the longitudinal CARDIA study. *American Journal of Respiratory and Critical Care Medicine*.

[32] Thuesen B. H., Husemoen L. L. N., Hersoug L.-G., Pisinger C., Linneberg A. (2009). Insulin resistance as a predictor of incident asthma-like symptoms in adults. *Clinical and Experimental Allergy*.

[33] Holguin F., Bleecker E. R., Busse W. W. (2011). Obesity and asthma: an association modified by age of asthma onset. *Journal of Allergy and Clinical Immunology*.

[34] Sutherland E. R., Goleva E., King T. S. (2012). Cluster analysis of obesity and asthma phenotypes. *PLoS ONE*.

[35] van Veen I. H., Ten Brinke A., Sterk P. J., Rabe K. F., Bel E. H. (2008). Airway inflammation in obese and nonobese patients with difficult-to-treat asthma. *Allergy*.

[36] Lessard A., Turcotte H., Cormier Y., Boulet L.-P. (2008). Obesity and asthma: a specific phenotype?. *Chest*.

[37] Sutherland T. J. T., Cowan J. O., Young S. (2008). The association between obesity and asthma: interactions between systemic and airway inflammation. *The American Journal of Respiratory and Critical Care Medicine*.

[38] Todd D. C., Armstrong S., D'Silva L., Allen C. J., Hargreave F. E., Parameswaran K. (2007). Effect of obesity on airway inflammation: a cross-sectional analysis of body mass index and sputum cell counts. *Clinical and Experimental Allergy*.

[39] Asthma S., Desai D., Newby C. (2013). Elevated sputum interleukin-5 and submucosal eosinophilia in obese individuals with. *American Journal of Respiratory and Critical Care Medicine*.

[40] Moore W. C., Hastie A. T., Li X. (2014). Sputum neutrophil counts are associated with more severe asthma phenotypes using cluster analysis. *Journal of Allergy and Clinical Immunology*.

[41] van Huisstede A., Rudolphus A., van Schadewijk A. (2014). Bronchial and systemic inflammation in morbidly obese subjects with asthma: a biopsy study. *The American Journal of Respiratory and Critical Care Medicine*.

[42] Visser M., Bouter L. M., McQuillan G. M., Wener M. H., Harris T. B. (1999). Elevated C-reactive protein levels in overweight and obese adults. *Journal of the American Medical Association*.

[43] Forsythe L. K., Wallace J. M. W., Livingstone M. B. E. (2008). Obesity and inflammation: the effects of weight loss.. *Nutrition Research Reviews*.

[44] Bulló M., García-Lorda P., Megias I., Salas-Salvadó J. (2003). Systemic inflammation, adipose tissue tumor necrosis factor, and leptin expression. *Obesity Research*.

[45] Wood L. G., Garg M. L., Gibson P. G. (2011). A high-fat challenge increases airway inflammation and impairs bronchodilator recovery in asthma. *Journal of Allergy and Clinical Immunology*.

[46] Sood A., Shore S. A. (2013). Adiponectin, leptin, and resistin in asthma: basic mechanisms through population studies. *Journal of Allergy*.

[47] Periyalil H. A., Gibson P. G., Wood L. G. (2013). Immunometabolism in obese asthmatics: are we there yet?. *Nutrients*.

[48] Fernandez-Boyanapalli R., Goleva E., Kolakowski C. (2013). Obesity impairs apoptotic cell clearance in asthma. *Journal of Allergy and Clinical Immunology*.

[49] Fu J.-J., Baines K. J., Wood L. G., Gibson P. G. (2013). Systemic inflammation is associated with differential gene expression and airway neutrophilia in asthma. *OMICS*.

[50] Baines K. J., Backer V., Gibson P. G., Powel H., Porsbjerg C. M. (2015). Impaired lung function is associated with systemic inflammation and macrophage activation. *European Respiratory Journal*.

[51] Wood L. G., Baines K. J., Fu J., Scott H. A., Gibson P. G. (2012). The neutrophilic inflammatory phenotype is associated with systemic inflammation in asthma. *Chest*.

[52] Specjalski K., Chełmińska M., Jassem E. (2015). YKL-40 protein correlates with the phenotype of asthma. *Lung*.

[53] Sideleva O., Suratt B. T., Black K. E. (2012). Obesity and asthma: an inflammatory disease of adipose tissue not the airway. *The American Journal of Respiratory and Critical Care Medicine*.

[54] Sutherland T. J. T., Sears M. R., McLachlan C. R., Poulton R., Hancox R. J. (2009). Leptin, adiponectin, and asthma: findings from a population-based cohort study. *Annals of Allergy, Asthma and Immunology*.

[55] Canöz M., Erdenen F., Uzun H., Muderrisoglu C., Aydin S. (2008). The relationship of inflammatory cytokines with asthma and obesity. *Clinical and Investigative Medicine*.

[56] Holguin F., Rojas M., Brown L. A., Fitzpatrick A. M. (2011). Airway and plasma leptin and adiponectin in lean and obese asthmatics and controls. *Journal of Asthma*.

[57] Berthon B. S., MacDonald-Wicks L. K., Gibson P. G., Wood L. G. (2013). Investigation of the association between dietary intake, disease severity and airway inflammation in asthma. *Respirology*.

[58] Leivo-Korpela S., Lehtimäki L., Vuolteenaho K. (2011). Adipokine resistin predicts anti-inflammatory effect of glucocorticoids in asthma. *Journal of Inflammation*.

[59] Sood A., Ford E. S., Camargo C. A. (2006). Association between leptin and asthma in adults. *Thorax*.

[60] Jang A.-S., Kim T.-H., Park J.-S. (2009). Association of serum leptin and adiponectin with obesity in asthmatics. *Journal of Asthma*.

[61] Newson R. B., Jones M., Forsberg B. (2014). The association of asthma, nasal allergies, and positive skin prick tests with obesity, leptin, and adiponectin. *Clinical & Experimental Allergy*.

[62] Ruhl C. E., Everhart J. E. (2001). Leptin concentrations in the United States: relations with demographic and anthropometric measures. *American Journal of Clinical Nutrition*.

[63] Newson R. B., Jones M., Forsberg B. (2014). The association of asthma, nasal allergies, and positive skin prick tests with obesity, leptin, and adiponectin. *Clinical and Experimental Allergy*.

[64] Sood A., Seagrave J., Herbert G. (2014). High sputum total adiponectin is associated with low odds for asthma. *Journal of Asthma*.

[65] Shore S. A., Schwartzman I. N., Mellema M. S., Flynt L., Imrich A., Johnston R. A. (2005). Effect of leptin on allergic airway responses in mice. *Journal of Allergy and Clinical Immunology*.

[66] Vernooy J. H. J., Ubags N. D. J., Brusselle G. G. (2013). Leptin as regulator of pulmonary immune responses: involvement in respiratory diseases. *Pulmonary Pharmacology and Therapeutics*.

[67] Bruno A., Pace E., Chanez P. (2009). Leptin and leptin receptor expression in asthma. *Journal of Allergy and Clinical Immunology*.

[68] Shin J. H., Kim J. H., Lee W. Y., Shim J. Y. (2008). The expression of adiponectin receptors and the effects of adiponectin and leptin on airway smooth muscle cells. *Yonsei Medical Journal*.

[69] Conus S., Bruno A., Simon H.-U. (2005). Leptin is an eosinophil survival factor. *Journal of Allergy and Clinical Immunology*.

[70] Ilmarinen P., Kankaanranta H. (2014). Eosinophil apoptosis as a therapeutic target in allergic asthma. *Basic and Clinical Pharmacology and Toxicology*.

[71] Sood A., Qualls C., Schuyler M. (2012). Low serum adiponectin predicts future risk for asthma in women. *American Journal of Respiratory and Critical Care Medicine*.

[72] Sood A., Dominic E., Qualls C. (2011). Serum adiponectin is associated with adverse outcomes of asthma in men but not in women. *Frontiers in Pharmacology*.

[73] Scott H. A., Gibson P. G., Garg M. L., Wood L. G. (2011). Airway inflammation is augmented by obesity and fatty acids in asthma. *European Respiratory Journal*.

[74] Pierre N., Deldicque L., Barbé C., Naslain D., Cani P. D., Francaux M. (2013). Toll-like receptor 4 knockout mice are protected against endoplasmic reticulum stress induced by a high-fat diet. *PLoS ONE*.

[75] Holguin F., Comhair S. A. A., Hazen S. L. (2013). An association between L-arginine/asymmetric dimethyl arginine balance, obesity, and the age of asthma onset phenotype. *American Journal of Respiratory and Critical Care Medicine*.

[76] Redington A. E. (2006). Modulation of nitric oxide pathways: therapeutic potential in asthma and chronic obstructive pulmonary disease. *European Journal of Pharmacology*.

[77] Foster M. W., Yang Z., Potts E. N., Foster W. M., Que L. G. (2011). *S*-nitrosoglutathione supplementation to ovalbumin-sensitized and -challenged mice ameliorates methacholine-induced bronchoconstriction. *American Journal of Physiology: Lung Cellular and Molecular Physiology*.

[78] North M. L., Grasemann H., Khanna N., Inman M. D., Gauvreau G. M., Scott J. A. (2013). Increased ornithine-derived polyamines cause airway hyperresponsiveness in a mouse model of asthma. *American Journal of Respiratory Cell and Molecular Biology*.

[79] Ilmarinen P., Moilanen E., Erjefält J. S., Kankaanranta H. (2015). The polyamine spermine promotes survival and activation of human eosinophils. *Journal of Allergy and Clinical Immunology*.

[80] Tam A., Morrish D., Wadsworth S., Dorscheid D., Man S. P., Sin D. D. (2011). The role of female hormones on lung function in chronic lung diseases. *BMC Women's Health*.

[81] Schatz M., Camargo C. A. (2003). The relationship of sex to asthma prevalence, health care utilization, and medications in a large managed care organization. *Annals of Allergy, Asthma & Immunology*.

[82] de Marco R., Locatelli F., Sunyer J., Burney P. (2000). Differences in incidence of reported asthma related to age in men and women: a retrospective analysis of the data of the European Respiratory Health Survey. *American Journal of Respiratory and Critical Care Medicine*.

[83] Romieu I., Fabre A., Fournier A. (2010). Postmenopausal hormone therapy and asthma onset in the E3N cohort. *Thorax*.

[84] Troisi R. J., Speizer F. E., Willett W. C., Trichopoulos D., Rosner B. (1995). Menopause, postmenopausal estrogen preparations, and the risk of adult- onset asthma: a prospective cohort study. *American Journal of Respiratory and Critical Care Medicine*.

[85] Lange P., Parner J., Prescott E., Ulrik C. S., Vestbo J. (2001). Exogenous female sex steroid hormones and risk of asthma and asthma-like symptoms: a cross sectional study of the general population. *Thorax*.

[86] Kos-Kudła B., Ostrowska Z., Marek B. (2000). Hormone replacement therapy in postmenopausal asthmatic women. *Journal of Clinical Pharmacy and Therapeutics*.

[87] Farha S., Asosingh K., Laskowski D. (2009). Effects of the menstrual cycle on lung function variables in women with asthma. *American Journal of Respiratory and Critical Care Medicine*.

[88] MacSali F., Svanes C., Sothern R. B. (2013). Menstrual cycle and respiratory symptoms in a general nordic-baltic population. *American Journal of Respiratory and Critical Care Medicine*.

[89] Mandhane P. J., Hanna S. E., Inman M. D. (2009). Changes in exhaled nitric oxide related to estrogen and progesterone during the menstrual cycle. *Chest*.

[90] Ligeiro De Oliveira A. P., Oliveira-Filho R. M., da Silva Z. L., Borelli P., Tavares de Lima W. (2004). Regulation of allergic lung inflammation in rats: Interaction between estradiol and corticosterone. *NeuroImmunoModulation*.

[91] Melgert B. N., Postma D. S., Kuipers I. (2005). Female mice are more susceptible to the development of allergic airway inflammation than male mice. *Clinical and Experimental Allergy*.

[92] Huber S. A., Pfaeffle B. (1994). Differential Th1 and Th2 cell responses in male and female BALB/c mice infected with coxsackievirus group B type 3. *Journal of Virology*.

[93] Cai Y., Zhou J., Webb D. C. (2012). Estrogen stimulates Th2 cytokine production and regulates the compartmentalisation of eosinophils during allergen challenge in a mouse model of asthma. *International Archives of Allergy and Immunology*.

[94] Tai P., Wang J., Jin H. (2008). Induction of regulatory T cells by physiological level estrogen. *Journal of Cellular Physiology*.

[95] Hellings P. W., Vandekerckhove P., Claeys R., Billen J., Kasran A., Ceuppens J. L. (2003). Progesterone increases airway eosinophilia and hyper-responsiveness in a murine model of allergic asthma. *Clinical and Experimental Allergy*.

[96] de Oliveira A. P. L., Domingos H. V., Cavriani G. (2007). Cellular recruitment and cytokine generation in a rat model of allergic lung inflammation are differentially modulated by progesterone and estradiol. *American Journal of Physiology—Cell Physiology*.

[97] Hamano N., Terada N., Maesako K.-I. (1998). Effect of female hormones on the production of IL-4 and IL-13 from peripheral blood mononuclear cells. *Acta Oto-Laryngologica, Supplement*.

[98] Verthelyi D., Klinman D. M. (2000). Sex hormone levels correlate with the activity of cytokine-secreting cells in vivo. *Immunology*.

[99] Araneo B. A., Dowell T., Diegel M., Daynes R. A. (1991). Dihydrotestosterone exerts a depressive influence on the production of interleukin-4 (IL-4), IL-5, and *γ*-interferon, but not IL-2 by activated murine T cells. *Blood*.

[100] Liva S. M., Voskuhl R. R. (2001). Testosterone acts directly on CD4^+^ T lymphocytes to increase IL-10 production. *Journal of Immunology*.

[101] Yamatomo T., Okano M., Ono T. (2001). Sex-related differences in the initiation of allergic rhinitis in mice. *Allergy*.

[102] Pergola C., Dodt G., Rossi A. (2008). ERK-mediated regulation of leukotriene biosynthesis by androgens: a molecular basis for gender differences in inflammation and asthma. *Proceedings of the National Academy of Sciences of the United States of America*.

[103] Melgert B. N., Oriss T. B., Qi Z. (2010). Macrophages: regulators of sex differences in asthma?. *American Journal of Respiratory Cell and Molecular Biology*.

[104] Mollerup S., Jørgensen K., Berge G., Haugen A. (2002). Expression of estrogen receptors *α* and *β* in human lung tissue and cell lines. *Lung Cancer*.

[105] Jain R., Ray J. M., Pan J.-H., Brody S. L. (2012). Sex hormone-dependent regulation of cilia beat frequency in airway epithelium. *American Journal of Respiratory Cell and Molecular Biology*.

[106] Wilson C. M., McPhaul M. J. (1996). A and B forms of the androgen receptor are expressed in a variety of human tissues. *Molecular and Cellular Endocrinology*.

[107] Banerjee S., Chambliss K. L., Mineo C., Shaul P. W. (2014). Recent insights into non-nuclear actions of estrogen receptor alpha. *Steroids*.

[108] Saint-Criq V., Rapetti-Mauss R., Yusef Y. R., Harvey B. J. (2012). Estrogen regulation of epithelial ion transport: implications in health and disease. *Steroids*.

[109] Tamaki M., Konno Y., Kobayashi Y. (2014). Expression and functional roles of G-protein-coupled estrogen receptor (GPER) in human eosinophils. *Immunology Letters*.

[110] Payne A. S., Freishtat R. J. (2012). Conserved steroid hormone homology converges on nuclear factor kappaB to modulate inflammation in asthma. *Journal of Investigative Medicine*.

[111] Gouon-Evans V., Pollard J. W. (2001). Eotaxin is required for eosinophil homing into the stroma of the pubertal and cycling uterus. *Endocrinology*.

[112] Rhen T., Grissom S., Afshari C., Cidlowski J. A. (2003). Dexamethasone blocks the rapid biological effects of 17beta-estradiol in the rat uterus without antagonizing its global genomic actions. *The FASEB Journal*.

[113] Ramos J. G., Varayoud J., Kass L. (2000). Estrogen and progesterone modulation of eosinophilic infiltration of the rat uterine cervix. *Steroids*.

[114] Coleman J. M., Naik C., Holguin F. (2012). Epithelial eotaxin-2 and eotaxin-3 expression: relation to asthma severity, luminal eosinophilia and age at onset. *Thorax*.

[115] Vieira V. J., Ronan A. M., Windt M. R., Tagliaferro A. R. (2005). Elevated atopy in healthy obese women. *American Journal of Clinical Nutrition*.

[116] Isidori A. M., Strollo F., Moré M. (2000). Leptin and aging: correlation with endocrine changes in male and female healthy adult populations of different body weights. *The Journal of Clinical Endocrinology & Metabolism*.

[117] Jamieson P. M., Nyirenda M. J., Walker B. R., Chapman K. E., Seckl J. R. (1999). Interactions between oestradiol and glucocorticoid regulatory effects on liver-specific glucocorticoid-inducible genes: possible evidence for a role of hepatic 11*β*-hydroxysteroid dehydrogenase type 1. *Journal of Endocrinology*.

[118] Katz P. P., Morris A., Julian L. (2010). Onset of depressive symptoms among adults with asthma: results from a longitudinal observational cohort. *Primary Care Respiratory Journal*.

[119] Nejtek V. A., Brown E. S., Khan D. A., Moore J. J., Van Wagner J., Perantie D. C. (2001). Prevalence of mood disorders and relationship to asthma severity in patients at an inner-city asthma clinic. *Annals of Allergy, Asthma & Immunology*.

[120] Rod N. H., Kristensen T. S., Lange P., Prescott E., Diderichsen F. (2012). Perceived stress and risk of adult-onset asthma and other atopic disorders: a longitudinal cohort study. *Allergy*.

[121] Coogan P. F., Yu J., O'Connor G. T. (2014). Experiences of racism and the incidence of adult-onset asthma in the black women's health study. *Chest*.

[122] Huovinen E., Kaprio J., Koskenvuo M. (2001). Asthma in relation to personality traits, life satisfaction, and stress: a prospective study among 11,000 adults. *Allergy*.

[123] Scott K. M., Von Korff M., Alonso J. (2008). Childhood adversity, early-onset depressive/anxiety disorders, and adult-onset asthma. *Psychosomatic Medicine*.

[124] Chida Y., Hamer M., Steptoe A. (2008). A bidirectional relationship between psychosocial factors and atopic disorders: a systematic review and meta-analysis. *Psychosomatic Medicine*.

[125] Boudreau M., Bacon S. L., Ouellet K., Jacob A., Lavoie K. L. (2014). Mediator effect of depressive symptoms on the association between BMI and asthma control in adults. *Chest*.

[126] Jiang M., Qin P., Yang X. (2014). Comorbidity between depression and asthma via immune-inflammatory pathways: a meta-analysis. *Journal of Affective Disorders*.

[127] Schiepers O. J. G., Wichers M. C., Maes M. (2005). Cytokines and major depression. *Progress in Neuro-Psychopharmacology and Biological Psychiatry*.

[128] Van Lieshout R. J., Bienenstock J., MacQueen G. M. (2009). A review of candidate pathways underlying the association between asthma and major depressive disorder. *Psychosomatic Medicine*.

[129] Wichers M. C., Maes M. (2004). The role of indoleamine 2,3-dioxygenase (IDO) in the pathophysiology of interferon-alpha-induced depression. *Journal of Psychiatry and Neuroscience*.

[130] Bakunina N., Pariante C. M., Zunszain P. A. (2015). Immune mechanisms linked to depression via oxidative stress and neuroprogression. *Immunology*.

[131] Iwata M., Ota K. T., Duman R. S. (2013). The inflammasome: pathways linking psychological stress, depression, and systemic illnesses. *Brain, Behavior, and Immunity*.

[132] Schroder K., Tschopp J. (2010). The inflammasomes. *Cell*.

[133] Zhu C.-B., Blakely R. D., Hewlett W. A. (2006). The proinflammatory cytokines interleukin-1beta and tumor necrosis factor-alpha activate serotonin transporters. *Neuropsychopharmacology*.

[134] Zhu C.-B., Lindler K. M., Owens A. W., Daws L. C., Blakely R. D., Hewlett W. A. (2010). Interleukin-1 receptor activation by systemic lipopolysaccharide induces behavioral despair linked to MAPK regulation of CNS serotonin transporters. *Neuropsychopharmacology*.

[135] Ramamoorthy S., Ramamoorthy J. D., Prasad P. D. (1995). Regulation of the human serotonin transporter by interleukin-*β*. *Biochemical and Biophysical Research Communications*.

[136] Kenis G., Maes M. (2002). Effects of antidepressants on the production of cytokines. *The International Journal of Neuropsychopharmacology*.

[137] Müller N., Schwarz M. J., Dehning S. (2006). The cyclooxygenase-2 inhibitor celecoxib has therapeutic effects in major depression: results of a double-blind, randomized, placebo controlled, add-on pilot study to reboxetine. *Molecular Psychiatry*.

[138] Guerra S., Sherrill D. L., Martinez F. D., Barbee R. A. (2002). Rhinitis as an independent risk factor for adult-onset asthma. *Journal of Allergy and Clinical Immunology*.

[139] Shaaban R., Zureik M., Soussan D. (2008). Rhinitis and onset of asthma: a longitudinal population-based study. *The Lancet*.

[140] Torén K., Olin A.-C., Hellgren J., Hermansson B.-A. (2002). Rhinitis increase the risk for adult-onset asthma—a Swedish population-based case-control study (MAP-study). *Respiratory Medicine*.

[141] Jarvis D., Newson R., Lotvall J. (2012). Asthma in adults and its association with chronic rhinosinusitis: the GA 2LEN survey in Europe. *Allergy*.

[142] Antó J. M., Sunyer J., Basagaña X. (2010). Risk factors of new-onset asthma in adults: a population-based international cohort study. *Allergy*.

[143] Guilbert T. W., Denlinger L. C. (2010). Role of infection in the development and exacerbation of asthma. *Expert Review of Respiratory Medicine*.

[144] Ciprandi G., Caimmi D., Del Giudice M. M., La Rosa M., Salpietro C., Marseglia G. L. (2012). Recent developments in united airways disease. *Allergy, Asthma and Immunology Research*.

[145] Ten Brinke A., Grootendorst D. C., Schmidt J. T. (2002). Chronic sinusitis in severe asthma is related to sputum eosinophilia. *Journal of Allergy and Clinical Immunology*.

[146] Hens G., Vanaudenaerde B. M., Bullens D. M. A. (2008). Sinonasal pathology in nonallergic asthma and COPD: ‘united airway disease’ beyond the scope of allergy. *Allergy*.

[147] Rondón C., Campo P., Herrera R. (2011). Nasal allergen provocation test with multiple aeroallergens detects polysensitization in local allergic rhinitis. *Journal of Allergy and Clinical Immunology*.

[148] Szczeklik A., Nizankowska E., Duplaga M. (2000). Natural history of aspirin-induced asthma. AIANE investigators. european network on aspirin-induced asthma. *European Respiratory Journal*.

[149] Steinke J. W., Borish L. (2015). Factors driving the aspirin exacerbated respiratory disease phenotype. *American Journal of Rhinology and Allergy*.

[150] Laidlaw T. M., Cutler A. J., Kidder M. S. (2014). Prostaglandin E2 resistance in granulocytes from patients with aspirin-exacerbated respiratory disease. *Journal of Allergy and Clinical Immunology*.

[151] Steinke J. W., Liu L., Huyett P., Negri J., Payne S. C., Borish L. (2013). Prominent role of IFN-*γ* in patients with aspirin-exacerbated respiratory disease. *Journal of Allergy and Clinical Immunology*.

[152] Shannon J., Ernst P., Yamauchi Y. (2008). Differences in airway cytokine profile in severe asthma compared to moderate asthma. *Chest*.

[153] Kim J.-H., Park B.-L., Cheong H. S. (2010). Genome-wide and follow-up studies identify CEP68 gene variants associated with risk of aspirin-intolerant asthma. *PLoS ONE*.

[154] Park S.-M., Park J. S., Park H.-S., Park C.-S. (2013). Unraveling the genetic basis of aspirin hypersensitivity in asthma beyond arachidonate pathways. *Allergy, Asthma and Immunology Research*.

[155] Van Zele T., Gevaert P., Watelet J.-B. (2004). *Staphylococcus aureus* colonization and IgE antibody formation to enterotoxins is increased in nasal polyposis. *Journal of Allergy and Clinical Immunology*.

[156] Erdogan T., Karakaya G., Kalyoncu A. F. (2014). Comorbid diseases in aspirin-exacerbated respiratory disease, and asthma. *Allergologia et Immunopathologia*.

[157] Bachert C., van Steen K., Zhang N. (2012). Specific IgE against *Staphylococcus aureus* enterotoxins: an independent risk factor for asthma. *The Journal of Allergy and Clinical Immunology*.

[158] Song W.-J., Chang Y.-S., Lim M.-K. (2014). Staphylococcal enterotoxin sensitization in a community-based population: a potential role in adult-onset asthma. *Clinical and Experimental Allergy*.

[159] Pastacaldi C., Lewis P., Howarth P. (2011). Staphylococci and staphylococcal superantigens in asthma and rhinitis: a systematic review and meta-analysis. *Allergy*.

[160] Barnes P. J. (2009). Intrinsic asthma: not so different from allergic asthma but driven by superantigens?. *Clinical & Experimental Allergy*.

[161] Bachert C., Gevaert P., Holtappels G., Johansson S. G. O., van Cauwenberge P. (2001). Total and specific IgE in nasal polyps is related to local eosinophilic inflammation. *Journal of Allergy and Clinical Immunology*.

[162] Bachert C., Zhang N., Holtappels G. (2010). Presence of IL-5 protein and IgE antibodies to staphylococcal enterotoxins in nasal polyps is associated with comorbid asthma. *The Journal of Allergy and Clinical Immunology*.

[163] Moore W. C., Bleecker E. R., Curran-Everett D. (2007). Characterization of the severe asthma phenotype by the national heart, lung, and blood institute's severe asthma research program. *Journal of Allergy and Clinical Immunology*.

[164] Hahn D. L., Schure A., Patel K., Childs T., Drizik E., Webley W. (2012). Chlamydia pneumoniae-specific IgE is prevalent in asthma and is associated with disease severity. *PLoS ONE*.

[165] Edwards M. R., Bartlett N. W., Hussell T., Openshaw P., Johnston S. L. (2012). The microbiology of asthma. *Nature Reviews Microbiology*.

[166] Hahn D. L. (1999). *Chlamydia pneumoniae*, asthma, and COPD: what is the evidence?. *Annals of Allergy, Asthma and Immunology*.

[167] Biscione G. L., Corne J., Chauhan A. J., Johnston S. L. (2004). Increased frequency of detection of *Chlamydophila pneumoniae* in asthma. *European Respiratory Journal*.

[168] Pasternack R., Huhtala H., Karjalainen J. (2005). *Chlamydophila* (*Chlamydia*) pneumoniae serology and asthma in adults: A longitudinal analysis. *Journal of Allergy and Clinical Immunology*.

[169] Park C.-S., Kim T.-B., Moon K. A. (2010). *Chlamydophila pneumoniae* enhances secretion of VEGF, TGF-beta and TIMP-1 from human bronchial epithelial cells under Th2 dominant microenvironment. *Allergy, Asthma and Immunology Research*.

[170] Kim T.-B., Moon K.-A., Lee K.-Y. (2009). Chlamydophila pneumoniae triggers release of CCL20 and vascular endothelial growth factor from human bronchial epithelial cells through enhanced intracellular oxidative stress and MAPK activation. *Journal of Clinical Immunology*.

[171] Rödel J., Woytas M., Groh A. (2000). Production of basic fibroblast growth factor and interleukin 6 by human smooth muscle cells following infection with *Chlamydia pneumoniae*. *Infection and Immunity*.

[172] Park C.-S., Lee Y. S., Kwon H.-S. (2012). Chlamydophila pneumoniae inhibits corticosteroidinduced suppression of metalloproteinase-9 and tissue inhibitor metalloproteinase-1 secretion by human peripheral blood mononuclear cells. *Journal of Medical Microbiology*.

[173] Sakurai-Komada N., Iso H., Koike K. A. (2014). Association between *Chlamydophila pneumoniae* infection and risk of coronary heart disease for Japanese: the JPHC study. *Atherosclerosis*.

[174] Holgate S. T. (2012). Innate and adaptive immune responses in asthma. *Nature Medicine*.

[175] Xiao C., Puddicombe S. M., Field S. (2011). Defective epithelial barrier function in asthma. *Journal of Allergy and Clinical Immunology*.

[176] Rezaee F., Georas S. N. (2014). Breaking barriers. New insights into airway epithelial barrier function in health and disease. *American Journal of Respiratory Cell and Molecular Biology*.

[177] Koppelman G. H., Meyers D. A., Howard T. D. (2009). Identification of PCDH1 as a novel susceptibility gene for bronchial hyperresponsiveness. *American Journal of Respiratory and Critical Care Medicine*.

[178] Bønnelykke K., Sleiman P., Nielsen K. (2014). A genome-wide association study identifies CDHR3 as a susceptibility locus for early childhood asthma with severe exacerbations. *Nature Genetics*.

[179] Wan Y. I., Shrine N. R. G., Soler Artigas M. (2012). Genome-wide association study to identify genetic determinants of severe asthma. *Thorax*.

[180] Balantič M., Rijavec M., Fležar M. (2013). A polymorphism in ORMDL3 is associated not only with asthma without rhinitis but also with chronic obstructive pulmonary disease. *Journal of Investigational Allergology and Clinical Immunology*.

[181] Gill M. A., Bajwa G., George T. A. (2010). Counterregulation between the Fc*ε*RI pathway and antiviral responses in human plasmacytoid dendritic cells. *Journal of Immunology*.

[182] Schamberger A. C., Mise N., Jia J. (2014). Cigarette smoke-induced disruption of bronchial epithelial tight junctions is prevented by transforming growth factor-*β*. *The American Journal of Respiratory Cell and Molecular Biology*.

[183] Gangl K., Reininger R., Bernhard D. (2009). Cigarette smoke facilitates allergen penetration across respiratory epithelium. *Allergy*.

[184] Sykes A., Edwards M. R., MacIntyre J. (2012). Rhinovirus 16-induced IFN-alpha and IFN-beta are deficient in bronchoalveolar lavage cells in asthmatic patients. *Journal of Allergy and Clinical Immunology*.

[185] Pritchard A. L., Carroll M. L., Burel J. G., White O. J., Phipps S., Upham J. W. (2012). Innate IFNs and plasmacytoid dendritic cells constrain Th2 cytokine responses to rhinovirus: a regulatory mechanism with relevance to asthma. *The Journal of Immunology*.

[186] Holtzman M., Patel D., Kim H., You Y., Zhang Y. (2011). Hypersusceptibility to respiratory viruses as a shared mechanism for asthma, chronic obstructive pulmonary disease, and cystic fibrosis. *The American Journal of Respiratory Cell and Molecular Biology*.

[187] Kim E. Y., Battaile J. T., Patel A. C. (2008). Persistent activation of an innate immune response translates respiratory viral infection into chronic lung disease. *Nature Medicine*.

[188] Trompette A., Divanovic S., Visintin A. (2009). Allergenicity resulting from functional mimicry of a Toll-like receptor complex protein. *Nature*.

[189] Hammad H., Chieppa M., Perros F., Willart M. A., Germain R. N., Lambrecht B. N. (2009). House dust mite allergen induces asthma via toll-like receptor 4 triggering of airway structural cells. *Nature Medicine*.

[190] Ilmarinen P., Hasala H., Sareila O., Moilanen E., Kankaanranta H. (2009). Bacterial DNA delays human eosinophil apoptosis. *Pulmonary Pharmacology and Therapeutics*.

[191] Månsson A., Cardell L.-O. (2009). Role of atopic status in Toll-like receptor (TLR)7- and TLR9-mediated activation of human eosinophils. *Journal of Leukocyte Biology*.

[192] József L., Khreiss T., Filep J. G. (2004). CpG motifs in bacterial DNA delay apoptosis of neutrophil granulocytes. *The FASEB Journal*.

[193] Busse P. J., Mathur S. K. (2010). Age-related changes in immune function: effect on airway inflammation. *Journal of Allergy and Clinical Immunology*.

[194] Lieberoth S., Backer V., Kyvik K. O. (2012). Intake of alcohol and risk of adult-onset asthma. *Respiratory Medicine*.

[195] Sillanaukee P., Strid N., Jousilahti P. (2001). Association of self-reported diseases and health care use with commonly used laboratory markers for alcohol consumption. *Alcohol and Alcoholism*.

[196] Sisson J. H. (2007). Alcohol and airways function in health and disease. *Alcohol*.

[197] Vally H., de Klerk N., Thompson P. J. (2000). Alcoholic drinks: important triggers for asthma. *Journal of Allergy and Clinical Immunology*.

[198] Domínguez-Santalla M. J., Vidal C., Viñuela J., Pérez L. F., González-Quintela A. (2001). Increased serum IgE in alcoholics: relationship with Th1/Th2 cytokine production by stimulated blood mononuclear cells. *Alcoholism: Clinical and Experimental Research*.

[199] Alonso M., Gomez-Rial J., Gude F., Vidal C., Gonzalez-Quintela A. (2012). Influence of experimental alcohol administration on serum immunoglobulin levels: contrasting effects on IgE and other immunoglobulin classes. *International Journal of Immunopathology and Pharmacology*.

[200] Vidal C., Armisén M., Domínguez-Santalla M. J., Gude F., Lojo S., González-Quintela A. (2002). Influence of alcohol consumption on serum immunoglobulin E levels in atopic and nonatopic adults. *Alcoholism: Clinical and Experimental Research*.

[201] Friedrich N., Husemoen L. L. N., Petersmann A., Nauck M., Völzke H., Linneberg A. (2008). The association between alcohol consumption and biomarkers of alcohol exposure with total serum immunoglobulin E levels. *Alcoholism: Clinical and Experimental Research*.

[202] Linneberg A., Petersen J., Nielsen N. H. (2003). The relationship of alcohol consumption to total immunoglobulin E and the development of immunoglobulin E sensitization: the Copenhagen Allergy study. *Clinical & Experimental Allergy*.

[203] Crews F. T., Bechara R., Brown L. A. (2006). Cytokines and alcohol. *Alcoholism: Clinical and Experimental Research*.

[204] Latif O., Peterson J. D., Waltenbaugh C. (2002). Alcohol-mediated polarization of type 1 and type 2 immune responses. *Frontiers in Bioscience*.

[205] Nakamura K., Nagata C., Fujii K. (2009). Cigarette smoking and the adult onset of bronchial asthma in Japanese men and women. *Annals of Allergy, Asthma and Immunology*.

[206] Torén K., Hermansson B.-A. (1999). Incidence rate of adult-onset asthma in relation to age, sex, atopy and smoking: a Swedish population-based study of 15,813 adults. *International Journal of Tuberculosis and Lung Disease*.

[207] Lajunen T. K., Jaakkola J. J. K., Jaakkola M. S. (2013). The synergistic effect of heredity and exposure to second-hand smoke on adult-onset asthma. *The American Journal of Respiratory and Critical Care Medicine*.

[208] Coogan P. F., Castro-Webb N., Yu J., O'Connor G. T., Palmer J. R., Rosenberg L. (2015). Active and passive smoking and the incidence of asthma in the Black Women's Health study. *American Journal of Respiratory and Critical Care Medicine*.

[209] Polosa R., Knoke J. D., Russo C. (2008). Cigarette smoking is associated with a greater risk of incident asthma in allergic rhinitis. *Journal of Allergy and Clinical Immunology*.

[210] Siroux V., Pin I., Oryszczyn M. P., Le Moual N., Kauffmann F. (2000). Relationships of active smoking to asthma and asthma severity in the EGEA study. Epidemiological study on the genetics and environment of asthma. *European Respiratory Journal*.

[211] Troisi R. J., Speizer F. E., Rosner B., Trichopoulos D., Willett W. C. (1995). Cigarette smoking and incidence of chronic bronchitis and asthma in women. *Chest*.

[212] Torén K., Ekerljung L., Kim J.-L. (2011). Adult-onset asthma in west Sweden—incidence, sex differences and impact of occupational exposures. *Respiratory Medicine*.

[213] Huovinen E., Kaprio J., Koskenvuo M. (2003). Factors associated to lifestyle and risk of adult onset asthma. *Respiratory Medicine*.

[214] Polosa R., Russo C., Caponnetto P. (2011). Greater severity of new onset asthma in allergic subjects who smoke: a 10-year longitudinal study. *Respiratory Research*.

[215] Westerhof G. A., Vollema E. M., Weersink E. J., Reinartz S. M., de Nijs S. B., Bel E. H. (2014). Predictors for the development of progressive severity in new-onset adult asthma. *Journal of Allergy and Clinical Immunology*.

[216] Aanerud M., Carsin A. E., Sunyer J. (2015). Interaction between asthma and smoking increases the risk of adult airway obstruction. *European Respiratory Journal*.

[217] Perret J. L., Dharmage S. C., Matheson M. C. (2013). The interplay between the effects of lifetime asthma, smoking, and atopy on fixed airflow obstruction in middle age. *American Journal of Respiratory and Critical Care Medicine*.

[218] O'Byrne P. M., Lamm C. J., Busse W. W., Tan W. C., Pedersen S., START Investigators Group (2009). The effects of inhaled budesonide on lung function in smokers and nonsmokers with mild persistent asthma. *Chest*.

[219] Kuschner W. G., D'Alessandro A., Wong H., Blanc P. D. (1996). Dose-dependent cigarette smoking-related inflammatory responses in healthy adults. *European Respiratory Journal*.

[220] Amin K., Ekberg-Jansson A., Löfdahl C.-G., Venge P. (2003). Relationship between inflammatory cells and structural changes in the lungs of asymptomatic and never smokers: a biopsy study. *Thorax*.

[221] Tam A., Wadsworth S., Dorscheid D., Man S. F. P., Sin D. D. (2011). The airway epithelium: more than just a structural barrier. *Therapeutic Advances in Respiratory Disease*.

[222] Hoffmann R. F., Zarrintan S., Brandenburg S. M. (2013). Prolonged cigarette smoke exposure alters mitochondrial structure and function in airway epithelial cells. *Respiratory Research*.

[223] Buro-Auriemma L. J., Salit J., Hackett N. R. (2013). Cigarette smoking induces small airway epithelial epigenetic changes with corresponding modulation of gene expression. *Human Molecular Genetics*.

[224] Spira A., Beane J., Shah V. (2004). Effects of cigarette smoke on the human airway epithelial cell transcriptome. *Proceedings of the National Academy of Sciences of the United States of America*.

[225] Walters M. S., De B. P., Salit J. (2014). Smoking accelerates aging of the small airway epithelium. *Respiratory Research*.

[226] Broekema M., Ten Hacken N. H. T., Volbeda F. (2009). Airway epithelial changes in smokers but not in ex-smokers with asthma. *The American Journal of Respiratory and Critical Care Medicine*.

[227] Thomson N. C., Chaudhuri R., Heaney L. G. (2013). Clinical outcomes and inflammatory biomarkers in current smokers and exsmokers with severe asthma. *Journal of Allergy and Clinical Immunology*.

[228] Kirkham P., Rahman I. (2006). Oxidative stress in asthma and COPD: antioxidants as a therapeutic strategy. *Pharmacology and Therapeutics*.

[229] Yang L.-L., Huang M.-S., Huang C.-C. (2011). The association between adult asthma and superoxide dismutase and catalase gene activity. *International Archives of Allergy and Immunology*.

[230] Larkin E. K., Gao Y.-T., Gebretsadik T. (2015). New risk factors for adult-onset incident asthma. A nested case-control study of host antioxidant defense. *American Journal of Respiratory and Critical Care Medicine*.

[231] Nagasaki T., Matsumoto H., Nakaji H. (2013). Smoking attenuates the age-related decrease in IgE levels and maintains eosinophilic inflammation. *Clinical and Experimental Allergy*.

[232] Nakamura Y., Miyata M., Ohba T. (2008). Cigarette smoke extract induces thymic stromal lymphopoietin expression, leading to T_H_2-type immune responses and airway inflammation. *Journal of Allergy and Clinical Immunology*.

[233] Young M. T., Sandler D. P., DeRoo L. A., Vedal S., Kaufman J. D., London S. J. (2014). Ambient air pollution exposure and incident adult asthma in a nationwide cohort of U.S. women. *The American Journal of Respiratory and Critical Care Medicine*.

[234] Jacquemin B., Siroux V., Sanchez M. (2015). Ambient air pollution and adult asthma incidence in six european cohorts (ESCAPE). *Environmental Health Perspectives*.

[235] Jacquemin B., Schikowski T., Carsin A. (2012). The role of air pollution in adult-onset asthma: a review of the current evidence. *Seminars in Respiratory and Critical Care Medicine*.

[236] Vossoughi M., Schikowski T., Vierkötter A. (2014). Air pollution and subclinical airway inflammation in the SALIA cohort study. *Immunity and Ageing*.

[237] Nel A. (2005). Atmosphere: air pollution-related illness: effects of particles. *Science*.

[238] Castro-Giner F., Künzli N., Jacquemin B. (2009). Traffic-related air pollution, oxidative stress genes, and asthma (ECHRS). *Environmental Health Perspectives*.

[239] Smith A. M. (2011). The epidemiology of work-related asthma. *Immunology and Allergy Clinics of North America*.

[240] Jeebhay M. F., Ngajilo D., Le Moual N. (2014). Risk factors for nonwork-related adult-onset asthma and occupational asthma: a comparative review. *Current Opinion in Allergy and Clinical Immunology*.

[241] Lemiere C., Nguyen S., Sava F., D'Alpaos V., Huaux F., Vandenplas O. (2014). Occupational asthma phenotypes identified by increased fractional exhaled nitric oxide after exposure to causal agents. *Journal of Allergy and Clinical Immunology*.

[242] Lummus Z. L., Wisnewski A. V., Bernstein D. I. (2011). Pathogenesis and disease mechanisms of occupational asthma. *Immunology and Allergy Clinics of North America*.

[243] Quirce S., Bernstein J. A. (2011). Old and new causes of occupational asthma. *Immunology and Allergy Clinics of North America*.

[244] Brooks S. M., Bernstein I. L. (2011). Irritant-induced airway disorders. *Immunology and Allergy Clinics of North America*.

[245] Muñoz X., Cruz M. J., Bustamante V., Lopez-Campos J. L., Barreiro E. (2014). Work-related asthma: diagnosis and prognosis of immunological occupational asthma and work-exacerbated asthma. *Journal of Investigational Allergology and Clinical Immunology*.

[246] Henneberger P. K., Redlich C. A., Callahan D. B. (2011). An official American thoracic society statement: work-exacerbated asthma. *The American Journal of Respiratory and Critical Care Medicine*.

[247] Lemière C., Boulet L.-P., Chaboillez S. (2013). Work-exacerbated asthma and occupational asthma: do they really differ?. *The Journal of Allergy and Clinical Immunology*.

[248] Siroux V., González J. R., Bouzigon E. (2014). Genetic heterogeneity of asthma phenotypes identified by a clustering approach. *European Respiratory Journal*.

